# Impact of Body Mass Index on Stroke in Extracorporeal Cardiopulmonary Resuscitation: Data from the Extracorporeal Life Support Organization Registry

**DOI:** 10.3390/jcm14072202

**Published:** 2025-03-24

**Authors:** Jin Kook Kang, Shi Nan Feng, Winnie L. Liu, Jiah Kim, Andrew Kalra, Patricia Brown, Christopher J. Wilcox, Daniel Brodie, Steven P. Keller, Bo Soo Kim, Glenn J. R. Whitman, Sung-Min Cho

**Affiliations:** 1 Division of Neuroscience Critical Care, Departments of Neurology, Neurosurgery, and Anesthesiology and Critical Care Medicine, Johns Hopkins University School of Medicine, Baltimore, MD 21205, USA; jkang71@jh.edu (J.K.K.); sfeng26@jh.edu (S.N.F.); wliu107@jhmi.edu (W.L.L.); jiah54.kim@gmail.com (J.K.); pbrown57@jhmi.edu (P.B.);; 2 Division of Cardiac Surgery, Department of Surgery, Johns Hopkins University School of Medicine, Baltimore, MD 21205, USA; nkalra3@jhmi.edu (A.K.); gwhitman@jhmi.edu (G.J.R.W.); 3 Sidney Kimmel Medical College, Thomas Jefferson University, Philadelphia, PA 19107, USA; 4 Department of Cardiovascular Intensive Care, Mercy Hospital of Buffalo, Buffalo, NY 14220, USA; cwilcox@buffalo.edu; 5 Division of Pulmonary and Critical Care Medicine, Department of Medicine, Johns Hopkins University School of Medicine, Baltimore, MD 21205, USA; danielbrodie@jhu.edu; 6 Department of Surgery and Perioperative Care, Dell Medical School, Austin, TX 78712, USA; bo.kim@austin.utexas.edu

**Keywords:** body mass index, extracorporeal cardiopulmonary resuscitation, stroke, ischemic, hemorrhagic, hospital mortality

## Abstract

**Objective:** We aimed to characterize the impact of body mass index (BMI) on stroke in patients receiving extracorporeal cardiopulmonary resuscitation (ECPR). **Methods:** We queried the Extracorporeal Life Support Organization registry for patients receiving ECPR (2020–2024). Patients were categorized into five BMI groups: underweight (<18.5 kg/m^2^), normal weight (18.5–24.9 kg/m^2^), overweight (25–29.9 kg/m^2^), class 1 obesity (30–34.9 kg/m^2^), and class 2 obesity or above (≥35 kg/m^2^). A generalized additive model (GAM) analysis was used to identify the BMI range with the greatest stroke risk. Multivariable regression was used to compare odds of stroke between standard BMI groups and normal weight. Propensity score matching was used to compare stroke and mortality between normal weight and the BMI group with the highest predicted stroke risk. **Results:** Of 6390 patients (median age = 57.5, 68.6% male), 470 (7.4%) had a stroke during ECMO support (4.5% ischemic; 3.4% hemorrhagic). A total of 9.6% (*n* = 131) of class 1 obesity patients experienced stroke compared with 6.6% (*n* = 111) of normal weight, 6.9% (*n* = 79) of class 2 obesity or above, 6.9% (*n* = 143) of overweight, and 5.4% (*n* = 6) of underweight patients (*p* = 0.01). The GAM analysis showed a highest predicted stroke risk for class 1 obesity patients (*n* = 1366), which was confirmed by multivariable regression (adjusted odds ratio (aOR) = 1.63, 95%CI = 1.01–2.62, *p* = 0.045). After propensity matching (*n* = 357 each), class 1 obesity was associated with ischemic (aOR = 2.01, 95%CI = 1.02–4.08, *p* = 0.047) but not hemorrhagic stroke. Odds of hospital mortality were higher in both class 1 and 2 obesity patients compared with normal weight. **Conclusions:** Class 1 obesity was associated with increased odds of ischemic but not hemorrhagic stroke compared with normal weight patients.

## 1. Introduction

Use of extracorporeal cardiopulmonary resuscitation (ECPR) with venoarterial extracorporeal membrane oxygenation (VA ECMO) has emerged as a potential rescue therapy for patients experiencing refractory cardiac arrest. Thus, understanding the potential risk factors influencing outcomes of ECPR in critically ill patient populations is of increasing importance. The prevalence of obesity among critically ill patients can be as high as 20%, and management of these patients poses specific challenges [1,2]. In fact, obesity was previously recognized as a relative contraindication for ECMO due to technical challenges as well as associations between obesity and increased morbidity and mortality due to chronic health conditions [3]. However, while some studies exist that suggest an increased risk of on-ECMO complications for patients with obesity, including bleeding, mortality, and cardiovascular, renal, and device-related complications [4,5,6], others indicate comparable or even favorable survival outcomes in select populations [7]. Given the complex interplay between obesity, cardiovascular physiology, and critical illness, further research is needed to clarify the risks and prognosis of this patient group, particularly in the setting of ECPR.

Currently, sparse literature exists on the impact of BMI on neurological outcomes, particularly in patients receiving ECPR. Elevated BMI can impact hemodynamics, vascular access, and systemic inflammation, all of which may increase the risk of neurological complications such as stroke in patients with obesity [8]. Obesity is also frequently associated with comorbid conditions that are well-established risk factors for stroke, including hypertension, diabetes, and dyslipidemia [9]. Additionally, ECPR may further amplify this risk due to the increased difficulty in achieving adequate perfusion, prolonged cannulation times, and potential challenges in anticoagulation management, such as altered drug pharmacokinetics or dosing uncertainties in patients with obesity. Notably, stroke is a common on-ECMO complication in ECPR patients, with one study using the Extracorporeal Life Support Organization (ELSO) registry finding that 7% of ECPR patients experienced ischemic stroke and 3% experienced hemorrhagic stroke [10]. Separately, elevated BMI has been independently associated with increased risk of stroke in multiple studies [11,12]. Furthermore, evidence exists to suggest an association between elevated BMI and adverse neurological outcomes, including stroke, in cardiac surgery recipients [13]. Existing literature on neurological outcomes in ECPR patients have focused on cerebral performance category (CPC) score, and these findings are mixed. One retrospective analysis investigating the impact of BMI on neurological outcomes in ECPR patients found that BMI was not associated with CPC score [14], while another study found that BMI ≥ 30 kg/m^2^ was associated with a worse CPC score [15]. However, the relationship between BMI and stroke in patients receiving ECPR has yet to be investigated.

Thus, our pilot study aims to fill this critical gap by characterizing the impact of BMI on stroke in patients receiving ECPR, with the hypothesis that elevated BMI is associated with increased risk of stroke. We investigated in-hospital mortality as a secondary outcome.

## 2. Methods

### 2.1. Data Source

We queried the Extracorporeal Life Support Organization (ELSO) registry, a voluntary international database that collects information on use, indications, and outcomes of ECMO support in adults and children from more than 50 countries [16]. The registry includes records on patient demographics, clinical characteristics, pre-ECMO conditions, hemodynamic and laboratory values collected before and during ECMO, outcomes data including survival, and complications data including neurologic complications during ECMO support. Diagnosis and medical history are reported according to the International Classification of Diseases (ICD) 9th edition (ICD-9) codes and ICD-10. Data from the ECPR addendum were not included in this study due to the high rate of missingness.

This retrospective observational cohort pilot study was approved by the Johns Hopkins Hospital Institutional Review Board with a waiver of informed consent (IRB00216321). This study is reported using Strengthening the Reporting of Observational Studies in Epidemiology (STROBE) guidelines [17].

### 2.2. Patients

Adults (age ≥ 18 years) receiving ECMO for ECPR as the indication from January 2020 through February 2024 were included in this study. Exclusion criteria included patients treated with non-ECPR-indicated VA ECMO, venovenous (VV) ECMO, pediatric patients, patients who received more than one ECMO run, and patients with missing sex, height, or weight information.

Patients were categorized into 5 standard BMI groups: underweight (<18.5 kg/m^2^), normal weight (18.5–24.9 kg/m^2^), overweight (25–29.9 kg/m^2^), class 1 obesity (30–34.9 kg/m^2^), and class 2 obesity or above (≥35 kg/m^2^). These classifications for BMI are currently in use by the both the National Institute of Health (NIH) and the World Health Organization (WHO) [18]. Patients with BMI > 60 were excluded from our study to remove extreme values of BMI, as this group represented a very small proportion of the overall cohort and may have introduced outliers that could disproportionately influence the analysis.

### 2.3. ELSO Data

For all included patients, we extracted the following information from the ELSO registry database: pre-ECMO demographic information, pre-ECMO clinical variables, pre-ECMO diagnoses, on-ECMO, laboratory values, on-ECMO clinical variables, and ECMO-associated morbidity and mortality, including neurologic complications. Our analysis included only patients who received ECPR support. The “ECMO circuit mechanical failure” variable included oxygenator failure, clots in the hemofilter or circuit component, cracks in pigtail connectors, circuit change, air in the circuit, cannula problems, pump failure, and tubing rupture. All arterial blood gas (ABG) values and pre-ECLS hemodynamics were measured no more than 6 h before ECLS. Twenty-four-hour ABG values were drawn between 18 and 30 h after ECLS start time. Renal replacement therapy occurred during ECMO support.

### 2.4. Outcomes and Definition

Our primary outcome was stroke, including both ischemic and hemorrhagic stroke, defined by the ELSO registry as central nervous system (CNS) infarction determined by ultrasound or computed tomography (CT); and intra- and extra-parenchymal CNS hemorrhage and intraventricular CNS hemorrhage, determined by CT or MRI methods. Anoxic brain injury was excluded. All strokes were diagnosed during ECMO support. In-hospital mortality was compared as a secondary outcome.

### 2.5. Statistical Analysis

Data on patient characteristics, clinical variables, and outcomes were summarized as the medians and interquartile range (IQR) for continuous variables and numbers and percentages for categorical variables. Baseline characteristics were compared using the chi-square or Fisher’s exact test for categorical variables. The Kruskal–Wallis test was utilized for continuous variables. Normality of variables was assessed using histogram visualization in addition to the Shapiro–Wilk test.

To explore the effect of BMI on stroke as a continuous variable and to identify the BMI range with the greatest predicted risk of stroke, we performed a generalized additive model (GAM) analysis. This enabled us to capture potential non-linear relationships between BMI and stroke risk, using smoothing splines while controlling for patient demographics and clinical variables. We then performed multivariable regression to assess the association between BMI categories and stroke risk, adjusting for clinically relevant covariates. The normal weight BMI category was treated as the reference group. Odds ratios (ORs) with 95% confidence intervals (CIs) were calculated.

We pre-selected covariates based on clinical relevance and prior literature. Covariates included in the multivariable logistic regression were age, sex, race, comorbidities including diabetes, heart failure, chronic kidney disease, hyperlipidemia, and chronic obstructive pulmonary disease, ECMO indication such as cardiovascular, infectious, and respiratory complications, hours on ECMO, pre-ECMO pH, pre-ECMO HCO_3_ (bicarbonate), pre-ECMO PaO_2_ (partial pressure of oxygen in arterial blood), PaO_2_ at 24 h, pump flow at 4 h, on-ECMO renal replacement therapy, and on-ECMO complications including circuit complications, gastrointestinal hemorrhage, seizure, arrythmia, and hemolysis. Collinearity between confounders was examined and subsequently judged to be problematic in the case of a variation inflation factor that was >5.

Propensity score matching (PSM) was used to balance baseline characteristics between patients with a BMI in the BMI category determined to have the highest risk of stroke (class 1 obesity) compared with normal weight patients according to GAM and multivariable regression analyses. Propensity scores were obtained by logistic regression. Participants were matched by age, sex, race, ECMO indication, hours on ECMO, pre-ECMO pH, pre-ECMO HCO_3_, pre-ECMO PaO_2_, PaO_2_ at 24 h, pump flow, on-ECMO renal replacement therapy, and on-ECMO complications including circuit complications, gastrointestinal hemorrhage, seizure, arrythmia, and hemolysis. The listwise deletion of cases with missing covariates or independent variables was used. Propensity score matching was conducted using one-to-one nearest neighbor matching without replacement with BMI 30 to 34.9 kg/m^2^ as the dependent variable within a caliper width of 0.2. Satisfactory matching was defined as an absolute value of the standardized mean difference (SMD) of <0.10. A *p*-value of <0.05 was considered statistically significant. All statistical analyses were performed using R Studio (R 4.1.2, 2022)**.**

## 3. Results

### 3.1. Study Population

Of 7873 patients recorded in the ELSO registry over the study period, there were 6390 patients (median age = 57.5, 68.6% male, median BMI = 28.1) who met the inclusion criteria of this analysis. Of these patients, 1690 (26.4%) were in the normal weight group, 2079 (32.5%) were in the overweight group, 1366 (21.4%) were in the class 1 obesity group, 1143 (17.9%) were in the class 2 obesity or above group, and 112 (1.75%) were in the underweight group. Table 1 presents the characteristics of the overall study population stratified by BMI category.

Compared with normal weight patients, class 1 obesity patients were of comparable age (58.0 years vs. 57.2 years, *p* < 0.001), while class 2 obesity or above patients were likely to be younger (55.2 years vs. 57.2 years, *p* < 0.001). Class 1 and 2 obesity patients spent less time on ECMO (67.5 and 68.0 h vs. 74.0 h, *p* = 0.077) and were also more likely to have comorbidities including diabetes, heart failure, hypertension, hyperlipidemia, and chronic kidney disease (*p* < 0.001). With respect to complications, class 1 and class 2 obesity or above patients required more renal replacement therapy (22.3% and 25.3% vs. 17.8%, *p* < 0.001, respectively) and had a higher prevalence of arrhythmia (18.9% and 16.6% vs. 10.9%, *p* < 0.001, respectively) (Table 1).

### 3.2. Stroke

Of the 6390 patients who met study inclusion criteria, 470 (7.4%) experienced stroke during ECMO support, including 289 (4.5%) patients who developed ischemic stroke and 216 (3.4%) patients who developed hemorrhagic stroke (Supplemental Table S1).

Overall, to 6.6% (*n* = 111) of normal weight patients that experienced stroke, compared with 6.9% (*n* = 143) of overweight patients, 9.6% (*n* = 131) of patients with class 1 obesity, 6.9% (*n* = 79) of patients with class 2 obesity or above, and 5.4% (*n* = 6) of underweight patients (*p* < 0.001) (Table 1). Stratified by subtype of stroke, 5.8% of class 1 obesity patients developed ischemic stroke (*n* = 79) compared with 4.1% (*n* = 70) of normal weight patients, and 4.2% (*n* = 58) developed hemorrhagic stroke compared with 3.1% (*n* = 53) of normal weight patients. Meanwhile, in the class 2 obesity or above group, 4.3% (*n* = 49) of patients developed ischemic stroke and 3.0% (*n* = 34) developed hemorrhagic stroke.

**Figure 1:**
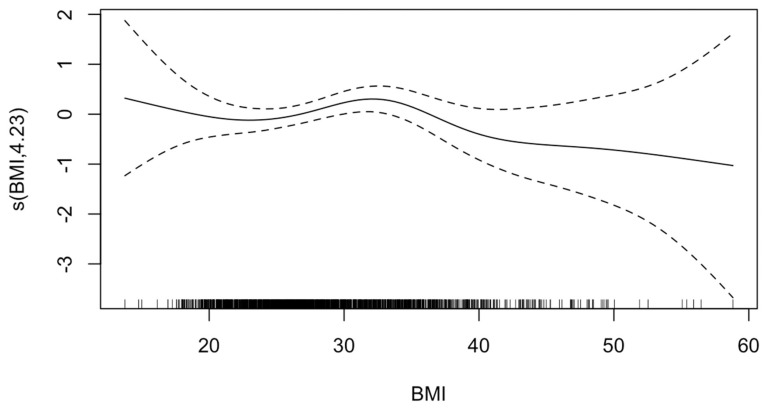
**Generalized additive model of effect of body mass index (BMI) on stroke.** The value on the *y*-axis indicates the contribution of BMI to the outcome after accounting for the smooth term in the model, representing the non-linear effect of BMI on risk of HIBI. The solid line represents the estimated effect, while the dashed lines show the confidence intervals around this effect. EDF, 4.23; *p*-value, 0.18. The effective degrees of freedom (EDF) of 4.23 indicates moderate flexibility in the model to capture the non-linear effects of BMI. The *p*-value of 0.18 indicates that there is insufficient evidence to reject the null hypothesis that BMI affects HIBI risk in a linear manner.

The GAM analysis adjusted for covariates revealed a non-linear relationship between BMI and stroke, suggesting a highest predicted risk of stroke (ischemic or hemorrhagic) in patients with class 1 obesity (EDF = 4.23; *p*-value = 0.18) (Figure 1). Multivariable regression confirmed that compared with normal weight patients, patients with class 1 obesity were 1.63 times as likely to experience stroke (95%CI = 1.01–2.62, *p* = 0.045). However, the odds were not significantly different for ischemic or hemorrhagic stroke in multivariable analyses stratified by stroke subtype. Notably, no significant differences were found in odds of stroke for any other BMI category compared with normal weight (Table 2).

In the propensity-matched cohort of 357 class 1 obesity patients and 357 normal weight patients, 14.3% (*n* = 51) of class 1 obesity patients experienced stroke compared with 9.2% (*n* = 131) of normal weight patients (Table 3). Class 1 obesity patients were 2.01 times as likely to experience ischemic stroke compared with normal weight patients (95%CI = 1.02–4.08, *p* = 0.047) (Table 4). However, no difference was observed in odds of hemorrhagic stroke. Supplemental Figure S1 shows the distribution of propensity scores for class 1 obesity versus normal weight patients.

### 3.3. In-Hospital Mortality

Of the entire study cohort (*n* = 6390), 4273 (66.9%) of patients died in-hospital (Supplemental Table S1). When stratified by BMI category, 61.5% (*n* = 1039) of normal weight patients died in-hospital compared with 65.6% (*n* = 1364) of overweight patients, 69.0% (*n* = 942) of class 1 obesity patients, 74.6% (*n* = 853) of class 2 obesity or above patients, and 67.0% (*n* = 75) of underweight patients (*p* < 0.001) (Table 1).

**Figure 2:**
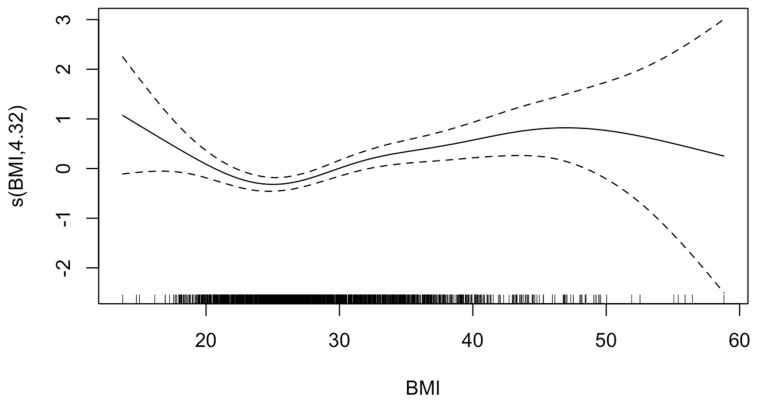
**Generalized additive model of effect of body mass index (BMI) on mortality.** The value on the *y*-axis indicates the contribution of BMI to the outcome after accounting for the smooth term in the model, representing the non-linear effect of BMI on risk of HIBI. The solid line represents the estimated effect, while the dashed lines show the confidence intervals around this effect. EDF, 4.32; *p*-value, <0.001. The effective degrees of freedom (EDF) of 4.32 indicates moderate flexibility in the model to capture the non-linear effects of BMI. The *p*-value of <0.001 indicates that there is sufficient evidence to reject the null hypothesis that BMI affects mortality risk in a linear manner.

The GAM analysis for hospital mortality risk revealed a significant relationship between both low and high values of BMI relative to normal weight (EDF = 4.32; *p*-value < 0.001) (Figure 2). Accordingly, multivariable regression revealed that compared with normal weight patients, patients with class 1 obesity were 1.54 times as likely to experience hospital mortality (95%CI = 1.11–2.15, *p* = 0.009), while patients with class 2 obesity or above were 2.01 times as likely to experience hospital mortality (95%CI = 1.39–2.94, *p* < 0.001) (Table 2).

In the propensity-matched cohort, 66.9% (*n* = 239) of patients with class 1 obesity died in-hospital compared with 56.6% (*n* = 202) patients of normal weight (Table 3). Patients with class 1 obesity were 1.84 times as likely to die in-hospital compared with normal weight patients (95%CI = 1.22–2.78, *p* = 0.004) (Table 4).

## 4. Discussion

In this large multicenter ELSO registry analysis, we found that compared with normal weight patients, the risk of stroke was greatest for patients in the class 1 obesity BMI category. An increased odds of ischemic stroke in class 1 obesity patients was supported by PSM and multivariable logistic regression. Interestingly, no statistically significant associations were found between BMI and stroke in other BMI categories compared with normal weight.

Our study suggests a non-linear relationship between BMI and stroke in this population, indicating that class 1 obesity patients receiving ECPR may experience the highest risk of stroke compared with patients in other BMI categories, including class 2 obesity or above. This finding raises important questions about whether specific physiological or technical factors uniquely predispose class 1 obesity patients to increased stroke risk. While we hypothesized that patients with class 1 obesity may represent a critical threshold where protective metabolic reserves are insufficient to offset the adverse effects of obesity-related inflammation, thus predisposing them to stroke; this reasoning remains speculative given the limitations of our data. Unmeasured technical factors could also play a role in increasing susceptibility to cerebral hypoperfusion or thromboembolic events. For example, higher BMI could preclude successful cannulation, potentially skewing the population with class 2 obesity or above towards younger, more robust patients with greater physiological reserves, who are likely to withstand aggressive interventions. Prior studies on BMI and VA ECMO outcomes have similarly highlighted complex associations, including one study that found worse mortality trends in patients with obesity but no statistically significant differences in complication rates [19]. Separately, that obesity was associated with ischemic but not hemorrhagic stroke in our study population may be explained by the role of neuronal and oligodendrocyte injury in altering the extracellular environment during stroke, triggering microglial activation, oxidative stress, and blood–brain barrier dysfunction, which can exacerbate ischemic injury without necessarily increasing hemorrhagic transformation [20].

Another potential explanation for the lower stroke risk observed in class 2 obesity or above patients is the competing risk of mortality. Given that class 2 obesity or above patients exhibited a higher odds of in-hospital mortality, it is possible that some patients in this group may have died before they could experience a stroke. Therefore, the reduced stroke risk in class 2 obesity or above patients may reflect a combination of both physiologic factors and the impact of competing risks such as death, which may have occurred before the onset of stroke or detection in some patients. Moreover, the challenges of imaging patients with higher BMIs while on ECMO may have resulted in underreporting of neurological complications including stroke, particularly among those with class 2 obesity or above. Thus, it remains possible that these data suggest that obesity may act as a general risk factor for stroke rather than indicate a significantly higher risk for class 1 obesity patients in particular. Ultimately, our findings highlight a critical need for future studies that explore the potential protective effects of higher BMI levels and how these effects may vary across obesity classes. Clinically, these findings highlight the need for tailored management strategies to minimize stroke risk and associated morbidity in patients with obesity receiving ECPR, including optimizing anticoagulation, ensuring adequate cerebral perfusion, minimizing cannulation complications, and enhancing early detection of neurological complications.

This study has a few limitations. First, given the retrospective, observational nature of the ELSO registry, we were inherently limited in our ability to infer causality from our conclusions. For example, rather than being a direct cause of stroke, obesity might contribute to technical challenges such as difficult cannulation and prolonged low-flow or no-flow times, thus indirectly leading to complications. Due to limitations in our dataset, we were also unable to examine time to stroke or account for death as a potential competing risk of stroke. Second, the ELSO registry gathers data on a voluntary basis, which may introduce potential for selection bias among participating centers. Third, higher BMI values may be underrepresented in our sample due to clinician biases when selecting candidates for cannulation, which could also affect the generalizability of our findings. Moreover, although we used standardized BMI classifications, variations in country-specific obesity definitions may introduce some heterogeneity in how obesity is categorized across different populations. Finally, the absence of detailed data on anticoagulation strategies during the ECMO run and time to cannulation represents another limitation of this study. To this degree, future studies incorporating more granular data, standardized reporting practices, and detailed clinical variables are necessary to refine and further our conclusions.

## 5. Conclusions

ECPR patients with class 1 obesity have increased odds of ischemic but not hemorrhagic stroke compared with normal weight. However, no statistically significant associations were found between BMI and any stroke type in other BMI categories.

## Figures and Tables

**Table 1:** Overall patient characteristics by BMI category.

	Normal Weight (REF) (*n* = 1690)	Overweight (*n* = 2079)	Class 1 Obesity (*n* = 1366)	≥Class 2 Obesity (*n* = 1143)	Underweight (*n* = 112)	*p*-Value
Age (years) (median [IQR])	57.2 [43.3, 66.5]	58.7 [48.2, 66.6]	58.0 [48.1, 66.1]	55.2 [44.4, 64.8]	50.9 [32.4, 65.6]	<0.001
Male sex (%)	1151 (68.1)	1538 (74.0)	969 (70.9)	674 (59.0)	54 (48.2)	<0.001
BMI (median [IQR])	23.0 [21.5, 24.1]	27.4 [26.1, 28.6]	32.2 [31.0, 33.5]	39.1 [36.8, 42.8]	17.8 [16.9, 18.1]	<0.001
Race (%)						<0.001
Black	167 (9.9)	184 (8.9)	195 (14.3)	216 (18.9)	17 (15.2)	<0.001
Hispanic	74 (4.4)	133 (6.4)	85 (6.2)	70 (6.1)	5 (4.5)	
Other	667 (39.5)	623 (30.0)	300 (22.0)	195 (17.1)	55 (49.1)	
White	782 (46.3)	1139 (54.8)	786 (57.5)	662 (57.9)	35 (31.2)	
Hours on ECMO (median [IQR])	74.0 [28.0, 139.0]	74.0 [27.0, 143.0]	67.5 [26.0, 138.75]	68.0 [22.0, 139.0]	66.5 [26.0, 144.75]	0.125
Comorbidities (%)						
Diabetes	93 (5.5)	160 (7.7)	183 (13.4)	158 (13.8)	3 (2.7)	<0.001
Heart failure	231 (13.7)	286 (13.8)	265 (19.4)	219 (19.2)	13 (11.6)	<0.001
Hypertension	168 (9.9)	290 (13.9)	260 (19.0)	210 (18.4)	9 (8.0)	<0.001
HLD	91 (5.4)	152 (7.3)	161 (11.8)	120 (10.5)	5 (4.5)	<0.001
COPD	36 (2.1)	37 (1.8)	30 (2.2)	27 (2.4)	4 (3.6)	0.614
CKD	74 (4.4)	91 (4.4)	96 (7.0)	83 (7.3)	4 (3.6)	<0.001
Diagnosis (%)						<0.001
Cardiovascular	1128 (66.7)	1403 (67.5)	885 (64.8)	676 (59.1)	77 (68.8)	
Chemical/Aspiration	0 (0.0)	3 (0.1)	0 (0.0)	0 (0.0)	1 (0.9)	
Hematologic	4 (0.2)	1 (0.0)	2 (0.1)	2 (0.2)	2 (1.8)	
Infectious	25 (1.5)	15 (0.7)	12 (0.9)	8 (0.7)	1 (0.9)	
LT complications	11 (0.7)	8 (0.4)	6 (0.4)	3 (0.3)	1 (0.9)	
Neoplastic disease	5 (0.3)	6 (0.3)	2 (0.1)	4 (0.3)	0 (0.0)	
Non-viral pneumonia	1 (0.1)	1 (0.0)	1 (0.1)	0 (0.0)	0 (0.0)	
Other	419 (24.8)	526 (25.3)	374 (27.4)	323 (28.3)	26 (23.2)	
PE/PH	33 (2.0)	64 (3.1)	59 (4.3)	93 (8.1)	3 (2.7)	
Respiratory disease	8 (0.4)	7 (0.3)	7 (0.5)	1 (0.1)	1 (0.9)	
Trauma/Burn	38 (2.2)	27 (1.3)	10 (0.7)	14 (1.2)	0 (0.0)	
ARDS/Acute respiratory failure	13 (0.8)	13 (0.6)	6 (0.4)	17 (1.5)	0 (0.0)	
Viral pneumonia	5 (0.3)	5 (0.2)	2 (0.1)	2 (0.2)	0 (0.0)	
Pre-ECMO blood gas and pump flow (median [IQR])						
pH	7.19 [7.01, 7.34]	7.18 [7.01, 7.32]	7.17 [7.01, 7.31]	7.16 [7.00, 7.29]	7.16 [6.99, 7.31]	0.118
PaO_2_	83.0 [56.0, 167]	82.0 [55.5, 156]	78.7 [56.0, 141]	76.5 [53.0, 134]	90.0 [54.5, 165]	0.148
PaCO_2_	46.5 [35.0, 65.0]	48.0 [36.0, 65.2]	49.2 [37.0, 66.7]	51.0 [38.0, 70.0]	50.0 [35.0, 61.0]	0.018
PaCO_2_ difference	−9.0 [−25.6, 3.0]	−9.6 [−28.0, 1.7]	−11.6 [−28.0, 0.0]	−14.2 [−32.5, 0.0]	−12.0 [−32.5, 5.9]	0.003
HCO_3_	18.0 [13.0, 23.0]	18.0 [13.4, 22.2]	18.0 [13.8, 22.0]	17.4 [14.0, 22.0]	16.5 [11.2, 22.5]	0.813
Pump flow	3.3 [2.8, 3.9]	3.5 [3.0, 4.1]	3.7 [3.1, 4.2]	3.9 [3.2, 4.4]	3.0 [2.4, 3.6]	<0.001
Complications (%)						
RRT required	300 (17.8)	402 (19.3)	305 (22.3)	293 (25.3)	22 (19.6)	<0.001
Hemolysis	52 (3.1)	72 (3.5)	55 (4.0)	52 (4.5)	1 (0.9)	0.115
Arrhythmia	185 (10.9)	319 (15.3)	258 (18.9)	190 (16.6)	11 (9.8)	<0.001
GI hemorrhage	61 (3.6)	95 (4.6)	45 (3.3)	50 (4.4)	0 (0.0)	0.053
Seizure	57 (3.4)	51 (2.5)	33 (2.4)	24 (2.1)	6 (5.4)	0.076
Stroke (%)	111 (6.6)	143 (6.9)	131 (9.6)	79 (6.9)	6 (5.4)	0.01
Ischemic stroke	70 (4.1)	88 (4.2)	79 (5.8)	49 (4.3)	3 (2.7)	0.134
Hemorrhagic stroke	53 (3.1)	68 (3.3)	58 (4.2)	34 (3.0)	3 (2.7)	0.371
Mortality (%)	1039 (61.5)	1364 (65.6)	942 (69.0)	853 (74.6)	75 (67.0)	<0.001

REF, reference group; IQR, interquartile range; BMI, body mass index; ECMO, extracorporeal membrane oxygenation; HLD, hyperlipidemia; COPD, chronic obstructive pulmonary disease; CKD, chronic kidney disease; PaO_2_, partial pressure of oxygen in arterial blood; PaCO_2_, partial pressure of carbon dioxide in arterial blood; HCO_3_, bicarbonate; RRT, renal replacement therapy; GI, gastrointestinal.

**Table 2:** Patient characteristics for normal weight and class 1 obesity matched cohort.

	Normal Weight (*n* = 357)	Obesity Class I (*n* = 357)	*p*-Value	SMD
BMI (median [IQR])	23.2 [21.6, 24.2]	32.2 [30.9, 33.5]	<0.001	6.069
Age years (median [IQR])	58.0 [47.3, 66.1]	57.7 [48.3, 67.2]	0.398	0.092
Male sex (%)	250 (70.0)	252 (70.6)	0.935	0.012
Race (%)				
Black	54 (15.1)	49 (13.7)	0.866	0.064
Hispanic	26 (7.3)	22 (6.2)		
Other	95 (26.6)	97 (27.2)		
White	182 (51.0)	189 (52.9)		
Hours on ECMO (median [IQR])	100.0 [60.0, 169.0]	94.0 [52.0, 161.0]	0.236	0.055
Comorbidities (%)				
Diabetes	35 (9.8)	38 (10.6)	0.805	0.028
Heart failure	79 (22.1)	85 (23.8)	0.656	0.040
Hypertension	53 (14.8)	63 (17.6)	0.361	0.076
COPD	8 (2.2)	8 (2.2)	1.000	<0.001
HLD	33 (9.2)	32 (9.0)	1.000	0.010
Diagnosis (%)	236 (66.1)	240 (67.2)	0.993	0.116
Cardiovascular diseases	236 (66.1)	240 (67.2)	0.993	0.116
Hematologic diseases	2 (0.6)	1 (0.3)		
Infectious diseases (Other)	4 (1.1)	5 (1.4)		
Lung transplant complications	1 (0.3)	2 (0.6)		
Non-viral pneumonia	1 (0.3)	1 (0.3)		
Other	93 (26.1)	89 (24.9)		
Pulmonary embolism/Pulmonary hypertension	9 (2.5)	10 (2.8)		
Respiratory diseases (Other)	2 (0.6)	2 (0.6)		
Trauma/Burn	3 (0.8)	4 (1.1)		
Unspecified ARDS/Acute respiratory failure	5 (1.4)	2 (0.6)		
Viral pneumonia	1 (0.3)	1 (0.3)		
Pre-ECMO blood gas and pump flow (median [IQR])				
pH	7.18 [7.03, 7.33]	7.20 [7.04, 7.32]	0.768	0.022
PaO_2_	82.0 [56.0, 167.2]	84.0 [57.0, 151.0]	0.739	0.010
PaCO_2_	47.0 [36.5, 63.7]	46.0 [36.0, 61.0]	0.289	0.087
PaCO_2_ difference	−9.9 [−26.0, 2.0]	−9.6 [−23.4, 1.6]	0.940	0.018
HCO_3_	18.0 [14.0, 23.5]	17.8 [13.7, 22.0]	0.261	0.049
Pump flow	3.5 [3.0, 4.1]	3.6 [3.1, 4.2]	0.225	0.076
Complications (%)				
RRT required	92 (25.8)	89 (24.9)	0.863	0.019
Hemolysis	23 (6.4)	25 (7.0)	0.881	0.022
Arrhythmia	60 (16.8)	73 (20.4)	0.249	0.094
GI hemorrhage	13 (3.6)	15 (4.2)	0.847	0.029
Seizure	15 (4.2)	12 (3.4)	0.695	0.044
HIBI	27 (7.6)	40 (11.2)	0.124	0.125
Stroke	33 (9.2)	51 (14.3)	0.048	0.157
Mortality (%)	202 (56.6)	239 (66.9)	0.006	0.214

REF, reference group; IQR, interquartile range; BMI, body mass index; ECMO, extracorporeal membrane oxygenation; HLD, hyperlipidemia; COPD, chronic obstructive pulmonary disease; PaO_2_, partial pressure of oxygen in arterial blood; PaCO_2_, partial pressure of carbon dioxide in arterial blood; HCO_3_, bicarbonate; RRT, renal replacement therapy; GI, gastrointestinal.

**Table 3:** Multivariable logistic regression for stroke and hospital mortality by BMI category.

Outcome (Ref: Normal Weight)	Odds Ratio	95%CI	*p*-Value
Stroke			
Overweight	1.15	0.75, 1.78	0.526
Class 1 Obesity	1.63	1.01, 2.62	0.045
≥Class 2 Obesity	0.86	0.48, 1.51	0.604
Underweight	1.67	0.46, 4.73	0.377
Ischemic Stroke			
Overweight	0.96	0.56, 1.65	0.878
Class 1 Obesity	1.73	0.98, 3.07	0.058
≥Class 2 Obesity	0.81	0.38, 1.63	0.560
Underweight	1.95	0.44, 6.23	0.310
Hemorrhagic Stroke			
Overweight	1.44	0.80, 2.65	0.918
Class 1 Obesity	1.10	0.55, 2.19	0.791
≥Class 2 Obesity	0.75	0.33, 1.65	0.478
Underweight	0.78	0.04, 4.29	0.013
Hospital Mortality			
Overweight	1.01	0.78, 1.32	0.918
Class 1 Obesity	1.54	1.11, 2.15	0.009
≥Class 2 Obesity	2.01	1.39, 2.94	<0.001
Underweight	3.01	1.30, 7.61	0.013

Covariates: age, sex, race, comorbidities including diabetes, heart failure, chronic kidney disease, hyperlipidemia, and chronic obstructive pulmonary disease, ECMO indication, hours on ECMO, pre-ECMO pH, pre-ECMO HCO_3_, pre-ECMO PaO_2_, PaO_2_ at 24 h, pump flow, on-ECMO renal replacement therapy, and on-ECMO complications including circuit complications, gastrointestinal hemorrhage, seizure, arrythmia, and hemolysis.

**Table 4:** Class 1 obesity and normal weight propensity-matched cohort multivariable logistic regression for stroke and hospital mortality.

Outcome (Ref: Normal Weight)	Odds Ratio	95%CI	*p*-Value
Stroke			
Class 1 Obesity	1.76	1.00, 3.13	0.050
Ischemic Stroke			
Class 1 Obesity	2.01	1.02, 4.08	0.047
Hemorrhagic Stroke			
Class 1 Obesity	0.84	0.35, 1.97	0.69
Hospital Mortality			
Class 1 Obesity	1.84	1.22, 2.78	0.004

Matched by race, sex, comorbidities including diabetes, heart failure, chronic kidney disease, hyperlipidemia, and chronic obstructive pulmonary disease, ECMO indication, hours on ECMO, pre-ECMO pH, pre-ECMO HCO_3_, pre-ECMO PaO_2_, PaO_2_ at 24 h, pump flow, on-ECMO renal replacement therapy, and on-ECMO complications including circuit complications, gastrointestinal hemorrhage, seizure, arrythmia, and hemolysis.

## Data Availability

Data sharing is restricted due to ethical restrictions and all data requests must be met through the ELSO.

## Supplementary Materials

The following supporting information can be downloaded at: https://www.mdpi.com/article/10.3390/jcm14072202/s1, Figure S1: Distribution of propensity scores for Class 1 Obesity vs. Normal Weight patients.; Table S1: Overall Patient Characteristics by BMI Category.
