# Supplementary material for: Impact of Body Mass Index on Stroke in Extracorporeal Cardiopulmonary Resuscitation: Data from the Extracorporeal Life Support Organization Registry
Source: J Clin Med. 2025 Mar 24;14(7):2202. doi: 10.3390/jcm14072202 (PMC11989509; doi:10.3390/jcm14072202)

SUPPLEMENTAL TABLE S1. Overall Patient Characteristics by BMI Category.

	Patients (n=6,390)
**Age (years) (median [IQR])**	57.50 [45.90, 66.10]
**Male Sex (%)**	4386 (68.6)
**BMI (median [IQR])**	28.1 [24.6, 33.0]
**Race (%)**	
Black	779 (12.2)
Hispanic	367 (5.7)
Other	1840 (28.8)
White	3404 (53.3)
**Hours on ECMO (median [IQR])**	72.0 [26.0, 141.0]
**Comorbidities (%)**	
Diabetes	597 (9.3)
Heart Failure	1014 (15.9)
Hypertension	937 (14.7)
HLD	529 (8.3)
COPD	134 (2.1)
CKD	348 (5.4)
**Complications (%)**	
RRT Required	1318 (20.6)
Hemolysis	232 (3.6)
Arrhythmia	963 (15.1)
GI Hemorrhage	251 (3.9)
Seizure	171 (2.7)
**Stroke (%)**	470 (7.4)
Ischemic Stroke	289 (4.5)
Hemorrhagic Stroke	216 (3.4)
**Mortality (%)**	4273 (66.9)

IQR, interquartile range. BMI, body mass index. ECMO, extracorporeal membrane oxygenation. HLD, hyperlipidemia. COPD, chronic obstructive pulmonary disease. CKD, chronic kidney disease. RRT, renal replacement therapy. GI, gastrointestinal.

SUPPLEMENTAL FIGURE S1. Distribution of propensity scores for Class 1 Obesity vs. Normal Weight patients.

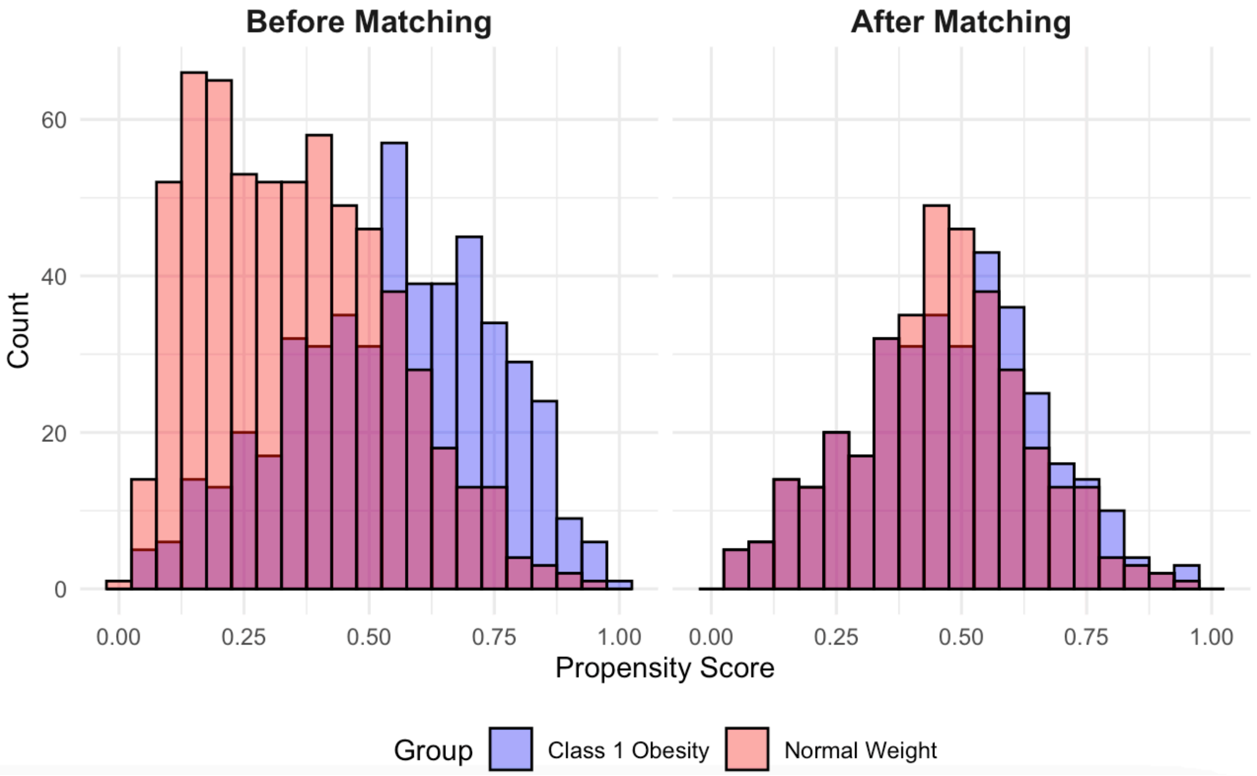

Supplement: Supplementary file 1 [file jcm-14-02202-s001.zip › jcm-3501700-supplementary.pdf]
